# Mapping Innovation Research in Organizations: A Bibliometric Analysis

**DOI:** 10.3389/fpsyg.2021.750960

**Published:** 2021-11-24

**Authors:** Renzhong Peng, Jingshuang Chen, Weiping Wu

**Affiliations:** ^1^School of Foreign Languages, Huazhong University of Science and Technology, Wuhan, China; ^2^School of Foreign Languages, Wuhan University of Technology, Wuhan, China

**Keywords:** innovation, creativity, organizations, bibliometric analysis, CiteSpace

## Abstract

This essay conducts a bibliometric study on innovation research in organizations within the three levels (i.e., individual, work team, and organizational) by using CiteSpace software to analyze 6,354 academic articles from the year 2000 to 2020 in four aspects: temporal distribution of published papers, scientific community (countries/regions/cited authors), intellectual structure (cited journals/cited references), and research hotspots. The research findings show that the total number and the growth rate of publications at the organizational level are far higher than the other two levels (individual and work team). The top three countries with the number of publications are United States, China, and United Kingdom. The top five highly cited authors are identified and listed from individual, work team, and organizational levels. *Academy of Management Journal* and *Academy of Management Review* are the top two highly cited journals at all three levels (i.e., individual, work team, and organizational levels). The most highly cited articles at the three levels are about topics of linking empowering leadership and employee creativity, team-level predictors of innovation at work, and organizational ambidexterity. The top three research hotspots are identified and listed from individual, work team, and organizational levels. These findings provide snapshots and comparisons of innovation research in management within the three levels (i.e., individual, work team, and organizational levels), which might be beneficial for researchers and scholars to understand and explore innovative behavior in organizations from a multilevel perspective.

## Introduction

Serving as a critical source of competitive advantage in organizations, innovation research has been deeply explored among researchers in management (Anderson et al., 2014). Research on innovation in organizations originates from the late 1960s, in which scholars conducted innovation research from an organizational perspective, especially in the healthcare industry, focusing on innovation diffusion (Walker, 1969) and centralization in organizations (Zaltman et al., 1973). From the early 1980s to the late 1990s, many studies expanded topics from the organizational level, such as innovation processes (Kimberly, 1981; Woodman et al., 1993; Amabile, 1997) and innovation determinants (complexity of structure, size, slack resources, and culture) (Rogers et al., 1983; Damanpour, 1991; West and Anderson, 1992), to the individual and work team levels involving personality characteristics (Barron and Harrington, 1981), motivation (Amabile, 1983), cognitive abilities (Kirton, 1999), team structure (West and Anderson, 1996), team climate (West and Anderson, 1996), and team processes (West, 1990). As innovation at the individual and work team levels has generally been studied in terms of the factors that determine creativity (Gupta et al., 2007), there is considerable overlap between research on innovation and creativity in organizations. Thus, the differences between creativity and innovation at these two levels are ignored in this study. Since the twenty-first century, innovation research has been undertaken from a multilevel perspective involving topics such as task and goal interdependence (Van der Vegt and Janssen, 2003), job characteristics (Baer et al., 2003), transformational leadership (Shin et al., 2012), social network (Shalley and Perry-Smith, 2008), and reflexivity climate (Jung et al., 2003).

Following a large number of articles on innovation in organizations at different levels, this article reviewed and synthesized these findings performed over the last decades through the use of a bibliometric approach. As the application of mathematics and statistical methods to the study of scientific publications (Leydesdorff, 1995), a bibliometric analysis is more objective and efficient than traditional qualitative analysis methods. There have been some previous bibliometric studies of innovation research, which mainly focused on reviewing some sub-topics of innovation, such as frugal innovation (Dangelo and Magnusson, 2021), open innovation (Randhawa et al., 2016), inclusive innovation (Mortazavi et al., 2021), and new product development (Marzi et al., 2021), or reviewing a specific journal related to innovation research, such as *Journal of Product Innovation Management* (Durisin et al., 2010; Antons et al., 2016; Sarin et al., 2018). To provide a comprehensive and systematic overview of innovation research in organizations, this study adopted CiteSpace, a widely used bibliometric mapping software to analyze the distribution of research publications, the scientific community (countries/regions/cited authors), intellectual structure (cited journals/cited references), and research hotspots. This study has involved a total of 6,354 articles (including the analysis of the related bibliographies, which correspond to approximately 234,000 references) published between 2000 and 2020.

As for the classification of innovation levels, despite the verified literature across management field of study, their meanings are basically the same, such as individual innovation and employee innovation, work team innovation, and work group innovation. Therefore, this study categorizes the three levels of innovation as the individual, work team, and organizational levels. The identified knowledge framework for innovation research at the three levels is beneficial for scholars to understand and explore the frontier of innovation research.

## Materials and Methods

### Research Questions

In the past two decades, innovation research in the field of management has emerged in an enormous amount, requiring scientific and systematic literature analysis. In doing so, the scientific community (countries/regions/cited authors), knowledge structure (cited journals/cited references), and research hotspots have been the main indicators for doing bibliometric analysis in the literature review (Pan et al., 2018). Accordingly, we investigated the following research questions:

RQ1. What is the distribution of research publications of innovation research at different levels (individual, work team, and organizational levels)?

RQ2. What is the scientific community of innovation research at different levels (individual, work team, and organizational levels)?

RQ3. What is the intellectual structure of innovation research at different levels (individual, work team, and organizational levels)?

RQ4. What are the research hotspots of innovation research at different levels (individual, work team, and organizational levels)?

### Data Collection

This article chose the subjects from articles in the Web of Science-Social Science Citation Index (SSCI) database. First, we preliminary searched the keywords “innovation, innovative, innovativeness, creative, and creativity” at different levels (individual, team, and organizational levels). The qualified keywords at the three levels were input as “employee, individual, work team, work group, and organizational.” For example, the search formula for innovation articles at the work team level was [TS = (*innovation* OR *innovative* OR *innovativeness* OR *creative* OR *creativity*) AND (*work team* OR *work group*)]. Second, during the data refining process, the time span was set from 2000 to 2020, the document type was set as “article,” the research area was set as “management,” and the language was set as “English.” Third, some articles not directly related to innovation or not mainly focused on innovation were removed through manual filtering. Meanwhile, the classification level was further determined through screening the abstracts of articles. Finally, we obtained a total of 6,354 articles, including 923 articles at the individual level, 1,205 articles at the work team level, and 4,226 articles at the organizational level.

### Statistical Analysis

The retrieved data were organized and analyzed by a set of different bibliometric analysis tools. First, the research publications on different levels of innovation research were displayed in a time-distributed manner by using the line chart. Second, the data from each level of innovation research were imported into CiteSpace software version 5.6.R2 for mapping countries (regions), cited authors, cited journals, cited references, and research hotspots so as to detect and visualize the research trends. In the knowledge map, the size of the node indicates the number of publications, and the number of concentric circles in the node indicates publication time. Moreover, the number of connections between one node and other nodes in the network is measured by centrality, which reflects the importance of that node (Chen, 2006). Finally, the research results were discussed.

## Results and Discussion

### Distribution of Research Publications

**FIGURE 1:**
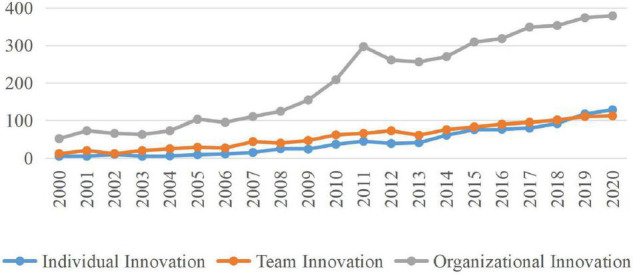
Annual publication volume of innovation research at different levels (2000–2020).

Figure 1 shows the diachronic changes in the volume of innovation research publications at different levels (individual, work team, and organizational levels) during 2000--2020. Although the number of published papers on innovation research at all three levels shows an overall increasing trend, the total number and the growth rate of publications at the organizational level are far higher than the other two levels (*N*_i_ = 923, *N*_t_ = 1,205, *N*_o_ = 4,226; *R*_i_ = 5.86%; *R*_t_ = 4.76%; *R*_o_ = 15.57%).^1^ As the pressure for organizational change has increased with progressing globalization and competition in the twenty-first century, the growing attention has been attached to organizational innovation (Poole and Van de Ven, 2004). After 6 years of steady development, the number of organizational innovation publications increased sharply from 2006 to 2011 (the number of publications increases from 95 to 297), which may result from a wave of mergers and acquisitions (M&As) around 2006 (Bhaskaran, 2006; Calipha et al., 2010). Through a slight decline in the number of organizational innovation articles from 2011 to 2013 (the number of publications increases from 297 to 256), the volume of articles rose constantly from 2013 to 2020 (the number of publications increases from 256 to 379), which reflects the fierce competition among enterprises from the change of the business environment and the tough challenge from new internet technology use and connection.

### Scientific Community

#### Publication Countries (Regions)

**FIGURE 2:**
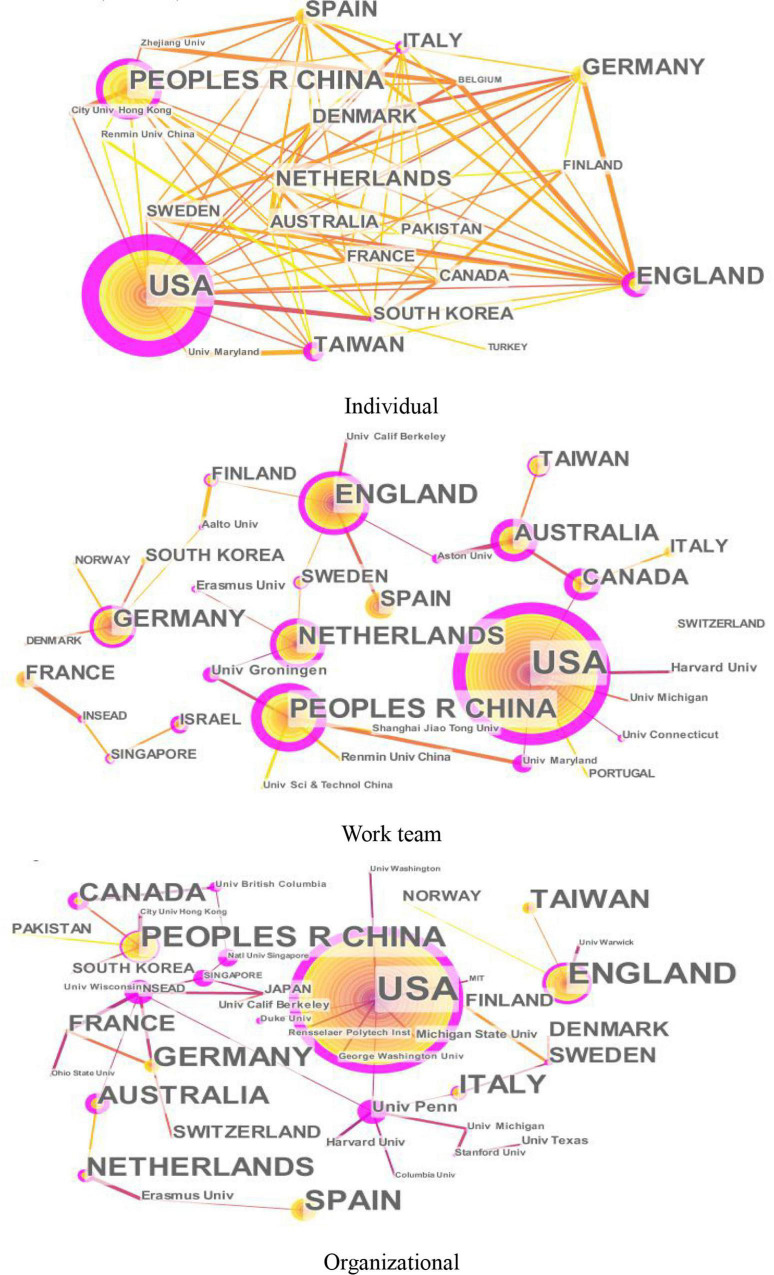
Knowledge maps of countries (regions) of innovation research at different levels.

Figure 2 shows the knowledge maps of publication countries (regions) of innovation research at different levels (individual, work team, and organizational levels), and Table 1 lists the top 10 publication countries (regions). As shown in Figure 2 and Table 1, the top three high publication countries at all the three levels of innovation research are United States, China, and United Kingdom.

**TABLE 1:** Top 10 publication countries (regions) of innovation research at different levels.

Level	Rank	Research countries (regions)	Number of publications	Centrality
Individual	1	United States	255	0.88
	2	China	151	0.24
	3	United Kingdom	92	0.36
	4	Spain	78	0.04
	5	Germany	72	0.02
	6	Taiwan	60	0.24
	7	Netherlands	59	0.08
	8	Italy	45	0.17
	9	Denmark	37	0.01
	10	Australia	32	0.04
Work team	1	United States	482	1.17
	2	China	152	0.92
	3	United Kingdom	149	0.46
	4	Netherlands	91	0.46
	5	Germany	89	0.25
	6	Australia	72	0.61
	7	Canada	51	0.66
	8	Spain	50	0.02
	9	Taiwan	42	0.12
	10	France	39	0.06
Organizational	1	United States	1,372	0.88
	2	United Kingdom	543	0.25
	3	China	406	0.19
	4	Spain	350	0.00
	5	Germany	261	0.06
	6	Italy	243	0.18
	7	Netherlands	230	0.34
	8	Taiwan	228	0.00
	9	Australia	195	0.40
	10	Canada	174	0.47

From Figure 2 and Table 1, United States was the highest-ranked country in the number of publications at the three levels, due to its four outstanding research institutions: University of Maryland, Harvard University, Michigan State University, and University of Pennsylvania. Specifically, the main contributors at the University of Maryland are Anil Gupta and Kathryn Bartol, both from the Center for Leadership, Innovation, and Change (CLIC), who are concerned with topics such as transformational leadership, individual skill development, team knowledge, and multiple-level innovation. At Harvard University, Teresa Amabile, Amy Edmondson, and Michael Tushman make prominent achievements in the work team and organizational innovation, whose research interests are team innovation process, psychological safety, strategic innovation, and open innovation. In addition, scholars such as Frederick Morgeson and Adam Grant from Michigan State University and the University of Pennsylvania mainly focus on leader–member exchange and intrinsic and prosocial motivations at the organizational level.

China ranks second at the individual and work team level and third at the organizational level, mainly contributed by three universities, namely, City University of Hong Kong, Renmin University of China, and Shanghai Jiao Tong University. Among these institutions, researchers such as Aurelia Mok, Kwok Leung, and Kwaku Atuahene-Gima from the City University of Hong Kong are interested in individual and organizational innovation, with their contributions in bicultural individuals’ creative styles, interpersonal harmony, and product innovation strategy. At Renmin University of China and Shanghai Jiao Tong University, researchers such as Jun Liu, Xiao-Hua Wang, and Ali Ahmad Bodla are dedicated to the work team level involving topics such as leadership style, social networks, and team diversity.

United Kingdom ranks third at the individual and work team level and second at the organizational level, primarily due to the contributions of three prominent institutions, namely, the University of Warwick, Aston University, and the University of Cambridge. At the University of Warwick, Jacky Swan from Innovation, Knowledge and Organizational Networks Research Unit (IKON) and Stephen Roper from the Department of Entrepreneurship & Innovation are the main contributors, with a focus on knowledge management, organizational learning, and networks of practice. At Aston University, Claudia Sacramento with her research team of the Entrepreneurship & Innovation has conducted various research on the determinants of effective team innovation, including bureaucratic practices and challenge stressors. At the University of Cambridge, Letizia Mortara from the Institute for Manufacturing and Shahzad Ansari from Cambridge Judge Business School have done some meaningful and influential research on open innovation and radical innovation in organizations.

#### Cited Authors

**FIGURE 3:**
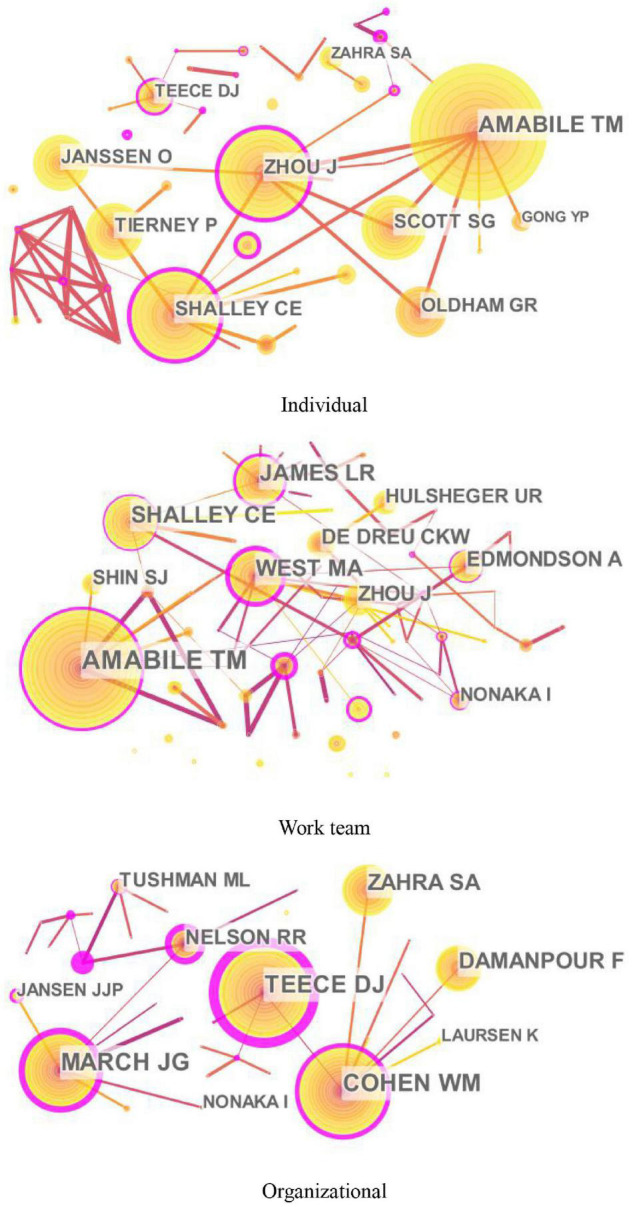
Knowledge maps of the cited authors of innovation research at different levels.

The knowledge maps of the highly cited authors of innovation research at different levels (individual, work team, and organizational levels) are shown in Figure 3, and Table 2 lists the top 10 highly cited authors.

**TABLE 2:** Top 10 highly cited authors of innovation research at different levels.

Level	Rank	Number of citation	Centrality	Cited author
Individual	1	248	0.10	Teresa Amabile
	2	159	0.44	Jing Zhou
	3	157	0.53	Christina Shalley
	4	114	0.00	Susanne Scott
	5	105	0.01	Onne Janssen
	6	104	0.04	Pamela Tierney
	7	95	0.03	Greg Oldham
	8	62	0.35	David Teece
	9	45	0.00	Shaker Zahra
	10	39	0.00	Yaping Gong
Work team	1	375	0.29	Teresa Amabile
	2	182	0.45	Michael West
	3	174	0.26	Lawrence James
	4	167	0.15	Christina Shalley
	5	112	0.10	Amy Edmondson
	6	105	0.06	Jing Zhou
	7	95	0.03	Carsten De Dreu
	8	79	0.00	Ute Hülsheger
	9	76	0.00	Shung Jae Shin
	10	73	0.17	Ikujiro Nonaka
Organizational	1	1,065	1.14	David Teece
	2	1,011	0.58	Wesley Cohen
	3	885	0.65	James March
	4	604	0.00	Shaker Zahra
	5	598	0.00	Fariborz Damanpour
	6	363	0.72	Richard Nelson
	7	245	0.18	Michael Tushman
	8	129	0.00	Ikujiro Nonaka
	9	122	0.26	Justin Jansen
	10	93	0.00	Keld Laursen

At the individual level, Teresa Amabile, Jing Zhou, Christina Shalley, Susanne Scott, and Onne Janssen are the top five highly cited scholars (see Figure 3 and Table 2). Teresa Amabile, from Harvard Business School, is the most highly cited author of individual innovation with the centrality of 0.10. By integrating individual creativity with the organizational work environment, she proposed the componential theory of organizational creativity and innovation and explored methods to evaluate creativity, motivation, and working environment through empirical research (e.g., Amabile, 1997). Her recent research investigated how everyday work life influenced individual creative performance, including factors such as identification with work, the meaning of work, life structure, key relationships, and participation in creative activities (Amabile, 2017). The second highly cited author is Jing Zhou from Rice University. Her major contributions are on developing the dual adjustment model of emotions for creativity in a supportive environment (George and Zhou, 2007). She is also dedicated to studying the interaction of personal and situational factors to facilitate or inhibit creativity, including job dissatisfaction, openness to experience, and conscientiousness. The third highly cited author is Christina Shalley from the Georgia Institute of Technology. Her main contribution is exploring the characteristics of work that affect innovative behavior, such as time deadlines and goals, work environment, and job complexity. Moreover, she proposed the centrality-creativity spiral model and emphasized the importance of both static and dynamic social networks to individual creativity (Perry-Smith and Shalley, 2003). Both the fourth highly cited author, Susanne Scott, and the fifth highly cited author, Onne Janssen, investigated determinants and path model of innovative behavior in the workplace, including factors such as leadership, individual problem-solving style, and job demands (Scott and Bruce, 1994; Janssen, 2000).

At the work team level, Teresa Amabile, Michael West, Lawrence James, Christina Shalley, and Amy Edmondson are the top five highly cited scholars (see Figure 3 and Table 2). Teresa Amabile ranks the first place with centrality (0.29) in the work team innovation. Her research interest has gradually expanded to the work team level since the twenty-first century, focusing on collaboration and helping in creative teams. Furthermore, she introduced four new constructs into the componential model of creativity and innovation in organizations: a sense of progress in creative idea development; the meaningfulness of the work to those carrying it out, affect, and synergistic extrinsic motivation (Amabile and Pratt, 2016). Both the second highly cited author, Michael West, and the fifth highly cited author, Amy Edmondson, focused on the innovation process of work team. The former made multiple contributions to the measurement of team climate, the Team Climate Inventory (TCI) (Anderson and West, 1998), and the team reflection theory, which shows how team reflection, planning, and action predict both team effectiveness and innovation in teams (West, 2000). The latter is mainly dedicated to explaining the definition and mechanism of psychological safety in innovation teams (Edmondson and Mogelof, 2006). Lawrence James ranks third with the number of citations. He made great contributions to the estimation methods in groups and laid the foundation for the measurement and explanation of team climate (James and Jones, 1974). The fourth highly cited author is Christina Shalley, who mainly contributes to detecting the relationship between the social network and team innovation: diverse personal ties outside of the team facilitate team creativity, especially outside ties with nationality-heterogeneous individuals and weak outside ties (Shalley and Perry-Smith, 2008).

At the organizational level, David Teece, Wesley Cohen, James March, Shaker Zahra, and Fariborz Damanpour are the top five highly cited scholars (see Figure 3 and Table 2). David Teece, the top-ranked scholar from the Haas School of Business, University of California, made great contributions to the definition and application of organizational dynamic capabilities, which is an important scholar of innovation performance (Teece, 2007). Moreover, other works done by David Teece deal with issues of facilitating innovation from business models and strategies and profiting from innovation. The second highly cited author is Wesley Cohen from the Fuqua School of Business, Duke. His main contribution is that he proposes the concept of “absorptive capacity” and explores its impact on related innovation activities, including basic research, the adoption and diffusion of innovations, and decisions to participate in cooperative R&D ventures (Cohen and Levinthal, 1990). His other efforts in exploring determinants of innovative activity and performance are also significant, including firm learning, market structure, and firm size. The third highly cited author is James March, who is a theoretical pioneer in the field of organizational innovation research. His major contributions are on organizational learning and decision-making, especially the delicate trade-off between exploration and exploitation (March, 1991). Shaker Zahra ranks fourth, whose research centered on international entrepreneurship, dynamic capabilities, and innovation strategy in organizations (Zahra and George, 2002). The fifth highly cited author is Fariborz Damanpour, who mainly contributes to exploring antecedents, processes, and outcomes of innovation in organizations, such as the relationship between organizational size and innovation (Damanpour, 1992), the characteristics of innovation adoption (Damanpour and Schneider, 2009), and the impact of different types of innovation on organizational performance (Damanpour et al., 1989).

### Intellectual Structure

#### Cited Journals

**FIGURE 4:**
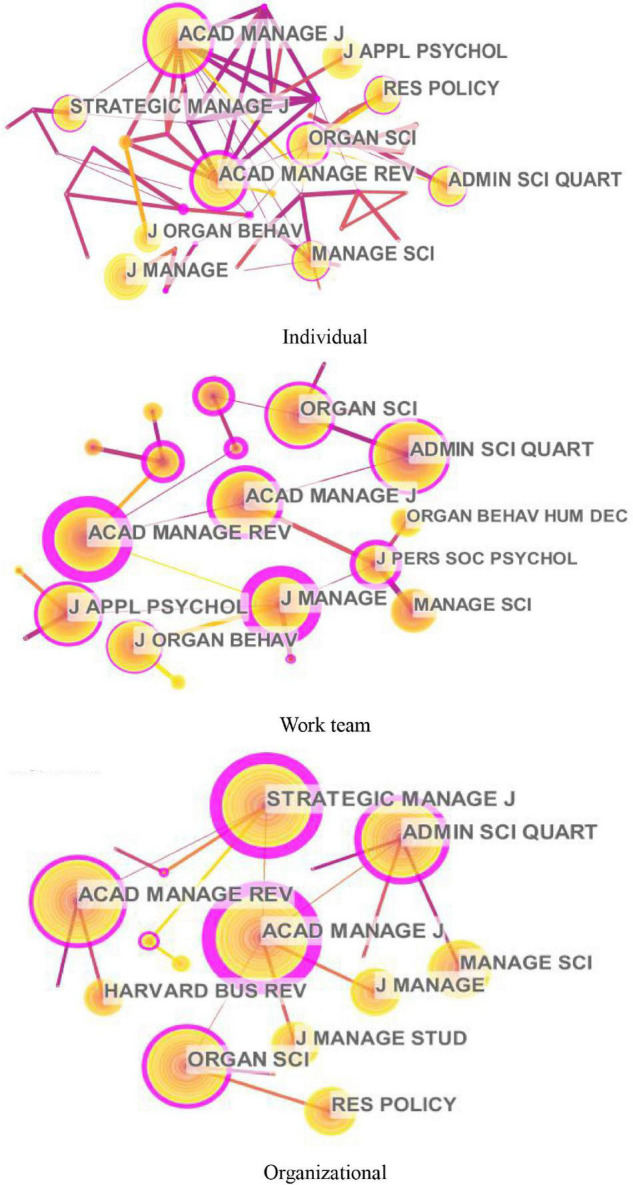
Knowledge maps of the cited journals of innovation research at different levels.

The knowledge maps of the highly cited journals of innovation research at different levels (individual, work team, and organizational levels) are shown in Figure 4, and Table 3 lists the top 10 highly cited journals. These active journals in Figure 4 and Table 3 indicate that innovation research involves a wide range of disciplines such as psychology, organizational behavior, organization science, and strategic management.

**TABLE 3:** Top 10 highly cited journals of innovation research at different levels.

Level	Rank	Number of citation	Centrality	Cited journal
Individual	1	617	0.49	*Academy of Management Journal*
	2	538	0.53	*Academy of Management Review*
	3	456	0.00	*Journal of Management*
	4	422	0.14	*Administrative Science Quarterly*
	5	422	0.38	*Organization Science*
	6	394	0.00	*Journal of Applied Psychology*
	7	390	0.16	*Research Policy*
	8	389	0.14	*Strategic Management Journal*
	9	378	0.15	*Management Science*
	10	284	0.00	*Journal of Organizational Behavior*
Work team	1	890	0.49	*Academy of Management Journal*
	2	834	1.05	*Academy of Management Review*
	3	763	0.40	*Administrative Science Quarterly*
	4	705	0.38	*Organization Science*
	5	691	0.29	*Journal of Applied Psychology*
	6	688	1.06	*Journal of Management*
	7	506	0.15	*Journal of Organizational Behavior*
	8	390	0.00	*Management Science*
	9	335	0.42	*Journal of Personality and Social Psychology*
	10	275	0.00	*Organizational Behavior and Human Decision Processes*
Organizational	1	3,053	1.46	*Academy of Management Journal*
	2	3,043	0.46	*Academy of Management Review*
	3	2,975	1.26	*Strategic Management Journal*
	4	2,806	0.46	*Organization Science*
	5	2,788	0.66	*Administrative Science Quarterly*
	6	2,282	0.00	*Management Science*
	7	2,052	0.00	*Research Policy*
	8	2,043	0.00	*Journal of Management*
	9	1,821	0.00	*Journal of Management Studies*
	10	1,693	0.00	*Harvard Business Review*

At the individual level, the top five highly cited journals are *Academy of Management Journal*, *Academy of Management Review*, *Journal of Management*, *Administrative Science Quarterly*, and *Organization Science* (see Figure 4 and Table 3). Among these journals, the top two highly cited journals, *Academy of Management Journal* (*AMJ*) and *Academy of Management Review* (*AMR*), cover a wide range of innovative topics from the macro-level to micro-level. However, *AMJ* attaches more importance to empirical research, while *AMR* mainly focuses on theoretical research. The third and fourth highly cited journals are *Journal of Management (JOM)* and *Administrative Science Quarterly* (*ASQ*), both of which are published by SAGE and cover all the three levels of innovative research. *JOM* involves various disciplines such as organizational behavior, entrepreneurship, and human resource management, while *ASQ* is committed to organizational studies. *Organization Science* (*OS*) ranks fifth with a centrality of 0.38, covering innovative topics such as fairness expectations, dynamic capabilities, and decision-making in individual innovative performance.

At the work team level, the top five highly cited journals are *Academy of Management Journal*, *Academy of Management Review*, *Administrative Science Quarterly*, *Organization Science*, and *Journal of Applied Psychology* (see Figure 4 and Table 3). The first two highly cited journals for work team innovation, *Academy of Management Journal* (*AMJ*) and *Academy of Management Review* (*AMR*), are the same as the top two cited journals at the individual level, which are comprehensive management journals covering topics such as team creative efficacy, team learning behavior, and the process of group creativity. The third and fourth highly cited journals are *Administrative Science Quarterly* (*ASQ*) (0.40 centrality) and *Organization Science* (*OS*) (0.38 centrality). As the top journals in the fields of management and organization theory, *ASQ* and *OS* both receive interdisciplinary research from organizational behavior, psychology, and sociology, involving team innovation topics such as structural dynamism, national diversity, and psychological safety. As a vital journal of the American Psychological Association, *Journal of Applied Psychology* (*JAP*) ranks fifth, investigating work team innovation from the perspective of team development, processes, and effectiveness.

At the organizational level, the top five highly cited journals are *Academy of Management Journal*, *Academy of Management Review*, *Strategic Management Journal*, *Organization Science*, and *Administrative Science Quarterly* (see Figure 4 and Table 3). The first two highly cited journals for organizational innovation, *Academy of Management Journal* (*AMJ*) and *Academy of Management Review* (*AMR*), are the same as the top two cited journals at the individual and work team level, covering topics such as organization structure, organizational policy, and human capital acquisition. The third highly cited journal is *Strategic Management Journal* (*SMJ*) with the centrality of 1.26. Specialized in strategic management, *SMJ* explores organizational innovation from the perspective of entrepreneurship, strategic resource allocation, and strategic decision processes. The other two journals are *Organization Science* (*OS*) and *Administrative Science Quarterly* (*ASQ*). In addition to the focus on work team innovation mentioned above, many innovation articles in *OS* and *ASQ* concern the organizational level involving topics such as organizational ambidexterity and collaborative innovation.

#### Cited References

**FIGURE 5:**
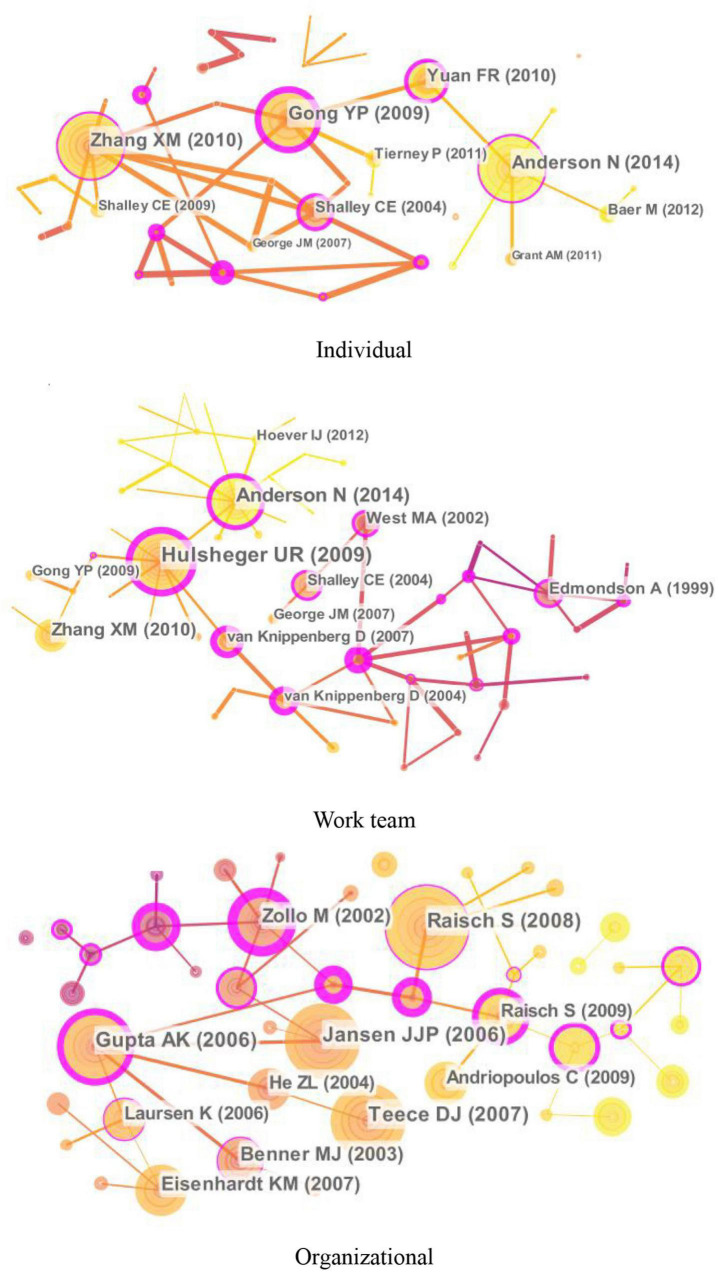
Knowledge maps of the cited references of innovation research at different levels.

The knowledge maps of the highly cited references of innovation research at different levels (individual, work team, and organizational levels) are shown in Figure 5, and Table 4 lists the top 10 highly cited references. These highly cited documents provide insights into theoretical knowledge, empirical evidence, and research pattern in innovation research at different levels.

**TABLE 4:** Top 10 highly cited references of innovation research at different levels.

Level	Rank	Number of citation	Cited references	Authors and years
Individual	1	54	Linking empowering leadership and employee creativity: The influence of psychological empowerment, intrinsic motivation, and creative process engagement	Zhang and Bartol, 2010
	2	54	Innovation and creativity in organizations: A state-of-the-science review, prospective commentary, and guiding framework	Anderson et al., 2014
	3	48	Employee learning orientation, transformational leadership, and employee creativity: The mediating role of employee creative self-efficacy	Gong et al., 2009
	4	34	Innovative behavior in the workplace: The role of performance and image outcome expectations	Yuan and Woodman, 2010
	5	25	The effects of personal and contextual characteristics on creativity: Where should we go from here?	Shalley et al., 2004
	6	17	Creative self-efficacy development and creative performance over time	Tierney and Farmer, 2011
	7	17	Putting creativity to work: The implementation of creative ideas in organizations	Baer, 2012
	8	14	Interactive effects of growth need strength, work context, and job complexity on self-reported creative performance	Shalley et al., 2009
	9	12	The necessity of others is the mother of invention: Intrinsic and prosocial motivations, perspective taking, and creativity	Grant and Berry, 2011
	10	11	Dual tuning in a supportive context: Joint contributions of positive mood, negative mood, and supervisory behaviors to employee creativity	George and Zhou, 2007
Work team	1	86	Team-level predictors of innovation at work: a comprehensive meta-analysis spanning three decades of research	Hülsheger et al., 2009
	2	71	Innovation and creativity in organizations: A state-of-the-science review, prospective commentary, and guiding framework	Anderson et al., 2014
	3	45	Linking empowering leadership and employee creativity: The influence of psychological empowerment, intrinsic motivation, and creative process engagement	Zhang and Bartol, 2010
	4	31	Psychological safety and learning behavior in work teams	Edmondson, 1999
	5	28	Sparkling fountains or stagnant ponds: An integrative model of creativity and innovation implementation in work groups	West, 2002
	6	26	Work group diversity	Van Knippenberg and Schippers, 2007
	7	25	The effects of personal and contextual characteristics on creativity: Where should we go from here?	Shalley et al., 2004
	8	20	Work group diversity and group performance: an integrative model and research agenda	Van Knippenberg et al., 2004
	9	19	9 Creativity in organizations	George, 2007
	10	17	Fostering team creativity: perspective taking as key to unlocking diversity’s potential	Hoever et al., 2012
Organizational	1	88	Organizational ambidexterity: Antecedents, outcomes, and moderators	Raisch and Birkinshaw, 2008
	2	81	Explicating dynamic capabilities: the nature and microfoundations of (sustainable) enterprise performance	Teece, 2007
	3	80	Exploratory innovation, exploitative innovation, and performance: Effects of organizational antecedents and environmental moderators	Jansen et al., 2006
	4	69	The interplay between exploration and exploitation	Gupta et al., 2006
	5	53	Deliberate learning and the evolution of dynamic capabilities	Zollo and Winter, 2002
	6	50	Exploitation, exploration, and process management: The productivity dilemma revisited	Benner and Tushman, 2003
	7	47	Exploration vs. exploitation: An empirical test of the ambidexterity hypothesis	He and Wong, 2004
	8	47	Exploitation-exploration tensions and organizational ambidexterity: Managing paradoxes of innovation	Andriopoulos and Lewis, 2009
	9	46	Organizational ambidexterity: Balancing exploitation and exploration for sustained performance	Raisch et al., 2009
	10	43	Open for innovation: the role of openness in explaining innovation performance among United Kingdom manufacturing firms	Laursen and Salter, 2006

Among the top five highly cited articles at the individual level (see Figure 5 and Table 4), the second highly cited article, *Innovation and creativity in organizations: A state-of-the-science review, prospective commentary, and guiding framework* (Anderson et al., 2014), and the fifth highly cited one, *The effects of personal and contextual characteristics on creativity*: *Where should we go from here* (Shalley et al., 2004), belong to literature review studies with traditional qualitative methods. The former provides insights into understanding how the range and variety of innovation research contribute to the various levels of analysis in organizations for scholars through a levels-of-analysis framework. The latter constructs a comprehensive model of employee creativity conducive to understanding the overall value and process of creative behaviors. The other three highly cited articles empirically test employee creativity with quantitative methods (e.g., questionnaires) and qualitative methods (e.g., focus group interview). Specifically, the first top highly cited one, *Linking empowering leadership and employee creativity: The influence of psychological empowerment, intrinsic motivation, and creative process engagement* (Zhang and Bartol, 2010), emphasizes the mediating role of empowering leadership on creativity via psychological empowerment, intrinsic motivation, which lays the foundation for further research and theory progress in investigating how empowering leadership can enhance innovative performance in organizations. The third top highly cited one, *Employee learning orientation, transformational leadership, and employee creativity: The mediating role of employee creative self-efficacy* (Gong et al., 2009), stresses the significant positive relationship among employee learning orientation, transformational leadership, and employee creativity. Their research findings not only provide evidence for researchers to confirm the practical value of studies on the antecedents of employee creativity but also offer constructive suggestions for managers to promote innovative performance by developing a learning orientation at the workplace. The fourth top highly cited one, *Innovative behavior in the workplace: The role of performance and image outcome expectations* (Yuan and Woodman, 2010), underlines the direct impact of outcome expectations of job performance and internal image of the organization on individual innovation behaviors. Their studies give substantial explanations for why employees are reluctant to innovate from the perspective of risks and benefits and offer solutions for managers to enhance employees’ willingness to innovate from two aspects of the relevant job requirements and the positive social recognition.

At the work team level (see Figure 5 and Table 4), the first top highly cited article, *Team-level predictors of innovation at work: a comprehensive meta-analysis spanning three decades of research* (Hülsheger et al., 2009), makes the first meta-analysis study to comprehensively analyze the antecedents of innovation at the team level, which is conducive to promoting the theory construction and detecting future research directions of innovation research in work teams. The second top highly cited one, *Innovation and creativity in organizations: A state-of-the-science review, prospective commentary, and guiding framework* (Anderson et al., 2014), provides multiple theoretical perspectives for researchers to further investigate work team innovation in organizations. The third top highly cited one, *Linking empowering leadership and employee creativity: The influence of psychological empowerment, intrinsic motivation, and creative process engagement* (Zhang and Bartol, 2010), provides guidance for cross-level innovation research by exploring the influence mechanism of team-level variables of empowering leadership on employee creativity. The fourth highly cited one, *Psychological safety and learning behavior in work teams* (Edmondson, 1999), indicates that the construction of team psychological safety is beneficial to understanding the collective learning process, proposes supplementary explanations for theories of team effectiveness, and lays an important theoretical foundation for examining the role of psychological safety in the innovation process. The fifth top highly cited one, *Sparkling fountains or stagnant ponds: An integrative model of creativity and innovation implementation in work groups* (West, 2002), establishes a basic model containing the dynamic and interactive process of work group innovation and provides constructive suggestions for supervisors to lead teams to innovate from the perspective of task characteristics, different support during the innovation process, and development of skills. Among them, some contribute a lot for the theoretical discussion on innovation research at the work team level and others enlighten scholars and researchers to further explore some influence mechanism of team-level variables and the construction of team psychological safety.

At the organizational level (see Figure 5 and Table 4), the first top highly cited article, *Organizational ambidexterity: Antecedents, outcomes, and moderators* (Raisch and Birkinshaw, 2008), provides a multidisciplinary knowledge base of organizational ambidexterity by identifying its antecedents, moderators, and outcomes, which could accelerate cross-fertilization across various disciplines and lay a theoretical foundation for studying the impact of organizational ambidexterity on organizational innovation. The second top highly cited one, *Explicating dynamic capabilities: the nature and microfoundations of (sustainable) enterprise performance* (Teece, 2007), identifies the most critical capabilities of management, entrepreneurial managerial capitalism, for enterprise sustainable development by integrating the strategy and innovation literature. The third top highly cited one, *Exploratory innovation, exploitative innovation, and performance: Effects of organizational antecedents and environmental moderators* (Jansen et al., 2006), empirically tests exploratory and exploitative innovation with quantitative methods (e.g., questionnaires). Jansen et al. (2006) offered empirical evidence for researchers and managers to understand the complicated process of coordinating the development of exploratory and exploitative innovation in ambidextrous organizations. The fourth top highly cited one, *The interplay between exploration and exploitation* (Gupta et al., 2006), puts forward the central issues of exploration and exploitation, including definitions and connotations, orthogonality vs. continuity, ambidexterity vs. punctuated equilibrium, and duality vs. specialization, which is beneficial to better understanding how complex organizational systems can gain competitive advantages and further studying on exploratory innovation and exploitative innovation. The fifth top highly cited one, *Deliberate learning and the evolution of dynamic capabilities* (Zollo and Winter, 2002), stresses the role of deliberate learning (including experience accumulation, knowledge articulation, and knowledge codification processes) in the mechanisms of dynamic capabilities development in organizations, which advances the understanding the functions of dynamic capabilities on long-run enterprise success and provides theoretical foundations and empirical inquiry for studying the impact of dynamic capabilities on innovations.

### Research Hotspots

**FIGURE 6:**
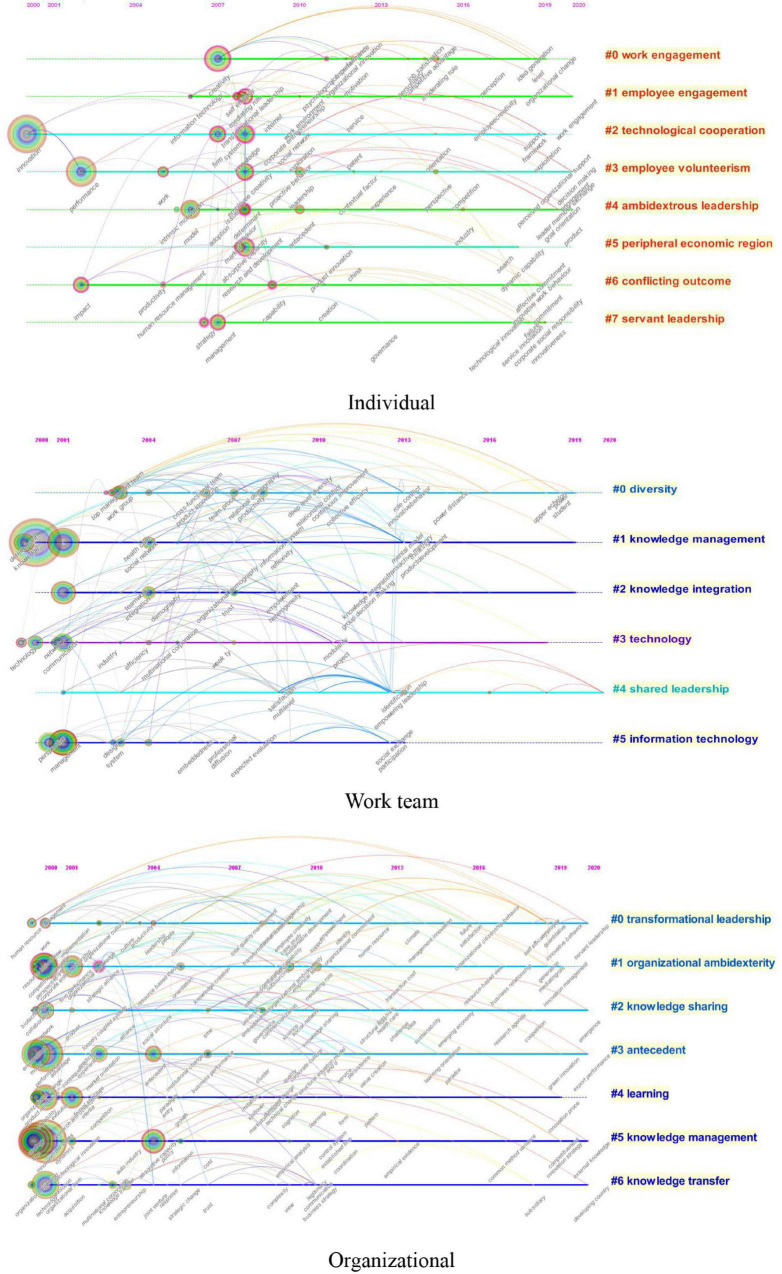
Knowledge maps of the research hotspots of innovation research at different levels (timeline view).

As keywords are the concentration and generalization of the core content of the literature, the analysis of keywords is beneficial to identify the research hotspots of a certain research field or discipline. After adopting the log-likelihood ratio (LLR) to cluster the keywords, 21 clusters were obtained (8 clusters at the individual level, 6 clusters at the work team level, and 7 clusters at the organizational level), and the detailed information of the clusters was listed in Table 5. These clusters were arranged along with horizontal timelines in Figure 6. As shown in Figure 6 and Table 5, the most frequent clustering labels in the three levels are about knowledge management process such as “knowledge management,” “knowledge integration,” “knowledge sharing,” and “knowledge transfer,” and leadership types such as “ambidextrous leadership,” “servant leadership,” “shared leadership,” and “transformational leadership.”

**TABLE 5:** Clusters of research hotspots of innovation research at different levels.

Level	Cluster ID	Name of cluster label	Cluster size
Individual	0	Work engagement	17
	1	Employee engagement	14
	2	Technological cooperation	13
	3	Employee volunteerism	12
	4	Ambidextrous leadership	11
	5	Peripheral economic region	11
	6	Conflicting outcome	10
	7	Servant leadership	9
Work team	0	Diversity	31
	1	Knowledge management	24
	2	Knowledge integration	24
	3	Technology	23
	4	Shared leadership	23
	5	Information technology	22
Organizational	0	Transformational leadership	35
	1	Organizational ambidexterity	27
	2	Knowledge sharing	27
	3	Antecedent	24
	4	Learning	23
	5	Knowledge management	22
	6	Knowledge transfer	21

At the individual level, the top three research hotspots are “work engagement,” “employee engagement,” and “technological cooperation” (see Figure 6 and Table 5). The first-ranked hotspot, “work engagement” (or “job engagement”), and the second-ranked hotspot, “employee engagement,” have the same meaning in the innovation literature, which refer to a measure of the vigor, dedication, and absorption experienced by the employee (Schaufeli et al., 2006). The connotation of work engagement can be roughly divided into three parts, namely, cognition, emotion, and behavior (Sundaray, 2011). Due to its positive impact on several individual and business outcomes such as individual productivity, business turnover, and managerial effectiveness (Blomme et al., 2015), work engagement has attracted scholars’ attention since 2006 (see the second line of individual innovation timelines in Figure 6). According to some major keywords in the first line of individual innovation timelines in Figure 6, scholars argued that work engagement was an important mediator in the relationship between several antecedents (e.g., job characteristics) and employee innovation (e.g., De Spiegelaere et al., 2014). Other researchers also emphasized the interaction between employee creativity and work engagement (e.g., Choi et al., 2015). Scholars’ enthusiasm for the research on work engagement continues until 2020 (see the second line of individual innovation timelines in Figure 6) by reviewing previous studies and exploring its impact in the new environment (e.g., organizational change). “Technological cooperation” ranks the third among the research hotspots of individual innovation, referring to the agreement for developing and executing a technological process to increase competitive advantage by combining or sharing skills and resources (Arranz and de Arroyabe, 2009). As its significant positive role in leveraging the capability of the company to adapt to a highly dynamic and complex environment (Alves et al., 2007), technological cooperation has attracted scholars’ attention since 2000 (see the third line of individual innovation timelines in Figure 6). Besides, studies on technological cooperation began to increase in 2007 and continued through 2019, mainly investigating the creative behaviors and processes of the employee in firms with technological cooperation, especially manufacturing firms (e.g., Alves et al., 2007).

At the work team level, the top three research hotspots are “diversity,” “knowledge management,” and “knowledge integration” (see Figure 6 and Table 5). Although “diversity” (ranks first) had been studied extensively by scholars in the late 1990s (e.g., Cady and Valentine, 1999), it gained renewed attention in 2003 when Van der Vegt and Janssen (2003) shed light on the joint impact of interdependence and group diversity on innovation (see the first line of work team innovation timelines in Figure 6). The popularity of diversity research continued to increase until 2011 and then gradually decreased. Some main keywords in Figure 6 show that the focus of diversity research has gradually shifted from demographic diversity or specific teams (e.g., top management team) to cultural diversity and the deep mechanisms of diversity impact on innovation. For example, Bouncken et al. (2016) conducted a longitudinal qualitative study and found that cultural diversity had a negative impact on innovation with difficulties arising in different working and communication styles, as well as conflicts in power distance. Both the second-ranked topic “knowledge management” and the third-ranked topic “knowledge integration” are important capabilities that promote team innovation. The concept of knowledge management is broader than knowledge integration as the former refers to the way to acquire, store, retrieve, share, and transfer all the information among members or across teams (Farr et al., 2003), while the latter refers to the synthesis of individual team members’ information through social interactions (Robert et al., 2008). As shown in Figure 6, the research boom of knowledge management appeared in 2000, and the popularity lasted until 2013 before it began to decline, covering topics related to the specific process of knowledge management (e.g., knowledge sharing and knowledge gathering) and types of innovative outcomes (e.g., radical innovation) (see the second line of work team innovation timelines in Figure 6). Besides, the research boom of knowledge integration was from 2001 to 2012, investigating the effect of knowledge integration on team innovation in a complicated and dynamic environment (see the third line of work team innovation timelines in Figure 6). For example, Koch (2011) proposed a conceptual framework of the relationship between knowledge integration and innovation and emphasized that innovation depended on efficient knowledge integration.

At the organizational level, the top three research hotspots are “transformational leadership,” “organizational ambidexterity,” and “knowledge sharing” (see Figure 6 and Table 5). “Transformational leadership” ranks the first among the research hotspots of organizational innovation, referring to behaviors of leaders who motivate employees to exceed expected levels of job performance and implement organizational goals (Sarros et al., 2008). According to Bass and Avolio (1995), transformational leadership consists of charismatic role modeling, individualized consideration, inspirational motivation, and intellectual stimulation. Although many studies have confirmed the positive effects of transformational leadership on individual creativity (e.g., Basu and Green, 1997), it was not until 2000 that the research on transformational leadership was extended to the organizational level (see the first line of organizational innovation timelines in Figure 6). One of the most influential studies in this period was that Jung et al. (2003) conducted an empirical survey in 32 Taiwanese firms, which proved a direct and positive link between transformational leadership and organizational climate. Recent studies have paid more attention to several emerging innovation outcomes (e.g., green innovation) and specific innovation processes (e.g., support for innovation). As a critical means to improve the competitive advantage of the company, “organizational ambidexterity,” the second-ranked hotspot, refers to the ability to balance two contradictory innovations, namely, exploratory innovation of existing knowledge and exploitative innovation of new possibilities (Raisch and Birkinshaw, 2008). There is an increasing interest in innovation research on organizational ambidexterity from 2000 to 2010 (see the second line of organizational innovation timelines in Figure 6), focusing on the general enablers and solutions of organizational ambidexterity, such as the integration and differentiation tactics (Andriopoulos and Lewis, 2009). After a decline from 2010 to 2017, research on organizational ambidexterity gained renewed attention in 2018, mainly investigating specific enablers and solutions, such as the use of high-performance work systems (Úbeda-García et al., 2018). The other research hotspot is “knowledge sharing,” a behavior or process of the exchange of employees’ knowledge and experiences at both individual and organizational levels (Lin, 2007). With the development of knowledge-based economy, knowledge sharing has been the focal point of innovation research between 2000 and 2014 (see the third line of organizational innovation timelines in Figure 6). Studies mainly examined two forms of knowledge sharing, knowledge donating and knowledge collecting, from the perspectives of antecedents and impacts. For example, Lin (2007) confirmed that enjoyment in helping others, knowledge self-efficacy, and top management support had a significant influence on knowledge-sharing processes; Wang and Wang (2012) argued that both explicit and tacit knowledge sharing had positive correlations with organizational innovation and performance.

## Conclusion

The study identifies the knowledge framework for innovation research from 2000 to 2020 within the three levels (i.e., individual, work team, and organizational levels), which includes the dimensions of temporal distribution, the scientific community, intellectual structure, and research hotspots. The main findings are the following. First, publication data indicate an overall increasing trend at all the three levels and the main position of research at the organization level among the three levels. Second, the common parts of the scientific community for innovation research at different levels include high number of articles published countries such as United States, China, and United Kingdom, and highly cited authors such as Teresa Amabile and Christina Shalley, indicating the possibility of the cross-level research at individual-team and team-organization interface. Third, the commonalities of the intellectual structure contain highly cited journals such as *AMJ*, *AMR*, *JOM*, *ASQ*, and *OS* and highly cited references about topics of linking empowering leadership and employee creativity, the effects of personal and contextual characteristics on creativity, and review of innovation and creativity in organizations, providing the theoretical and methodological basis, empirical examples and future directions for cross-level innovation research. Specifically, there are more influential empirical studies and literature reviews on the individual and work team levels, and influential studies on organizational innovation pay more attention to theoretical interpretation. Finally, research hotspots concerning the knowledge management process and leadership types are found to be studied on multiple levels. Through the analysis, scholars are provided with similarities and differences at all three levels of innovation research and then enhance a comprehensive understanding of innovation with the multilevel perspective.

There are inevitably some limitations in this study. On the one hand, this research only summarizes articles in the Web of Science-Social Science Citation Index database. Some other databases involving innovation research in organizations such as ProQuest One Business are ignored and can be included in future studies. On the other hand, although several manual screening criteria were set to filter articles, there may be subjective bias. Besides, further studies can be conducted with meta-analyses to build a multilevel innovation research mechanism model.

## Data Availability Statement

The original contributions presented in the study are included in the article/supplementary material, further inquiries can be directed to the corresponding author.

## Author Contributions

RP and JC participated in the whole process of writing the essay. WW gave the ideas and instructions on the whole manuscript. All authors contributed to the article and approved the submitted version.

## Conflict of Interest

The authors declare that the research was conducted in the absence of any commercial or financial relationships that could be construed as a potential conflict of interest.

## Publisher’s Note

All claims expressed in this article are solely those of the authors and do not necessarily represent those of their affiliated organizations, or those of the publisher, the editors and the reviewers. Any product that may be evaluated in this article, or claim that may be made by its manufacturer, is not guaranteed or endorsed by the publisher.

## Footnotes

1 *N*_i_, *N*_t_, and *N*_o_ represent the total number of publications at different levels (individual, work team, and organizational levels). *R*_i_, *R*_t_, and *R*_o_ represent the mean growth rate of publications at different levels (individual, work team, and organizational levels).
